# Understanding and Treating Niemann–Pick Type C Disease: Models Matter

**DOI:** 10.3390/ijms21238979

**Published:** 2020-11-26

**Authors:** Valentina Pallottini, Frank W. Pfrieger

**Affiliations:** 1 Biomedical and Technology Science Section, Department of Science, Roma Tre University, Viale Marconi 446, 00146 Rome, Italy; 2 Centre National de la Recherche Scientifique, Institut des Neurosciences Cellulaires et Intégratives, Université de Strasbourg, 8 Allée General Rouvillois, 67000 Strasbourg, France

**Keywords:** lysosomal disorder, cholesterol, transgenic, cell culture, induced pluripotent stem cells, neurodegeneration, *Drosophila*, zebrafish, *C. elegans*, feline

## Abstract

Biomedical research aims to understand the molecular mechanisms causing human diseases and to develop curative therapies. So far, these goals have been achieved for a small fraction of diseases, limiting factors being the availability, validity, and use of experimental models. Niemann–Pick type C (NPC) is a prime example for a disease that lacks a curative therapy despite substantial breakthroughs. This rare, fatal, and autosomal-recessive disorder is caused by defects in NPC1 or NPC2. These ubiquitously expressed proteins help cholesterol exit from the endosomal–lysosomal system. The dysfunction of either causes an aberrant accumulation of lipids with patients presenting a large range of disease onset, neurovisceral symptoms, and life span. Here, we note general aspects of experimental models, we describe the line-up used for NPC-related research and therapy development, and we provide an outlook on future topics.

## 1. Niemann–Pick Type C Disease

The prime purpose of biomedical research is to understand the molecular underpinnings of human diseases enabling the development of curative therapies. Unfortunately, these goals have been reached merely for a minuscule fraction of diseases. The large majority of ailments—affecting from just a handful of patients to millions worldwide—awaits a treatment [1,2,3]. There are numerous reasons for the slow progress such as rare occurrence, molecular complexity, and variability of symptoms. However, a decisive factor is the availability, quality, and use of experimental models [4,5,6,7,8,9,10,11,12,13].

**Figure 1:**
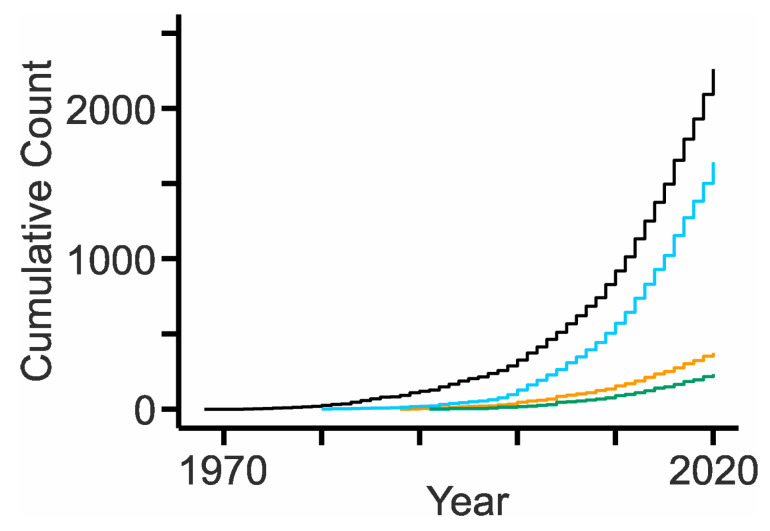
Growth of the NPC research field. Cumulative counts of publications obtained by Boolean queries in PubMed using the keywords “Niemann–Pick type c OR Niemann–Pick type c1 OR Niemann-Pick type c2 OR npc1 OR npc2” (Appendix A). Black and orange lines indicate original articles “(...) NOT review [pt]” and reviews [pt] “(...) AND review [pt]”, respectively. To retrieve publications more specifically related to NPC, we restricted the query to titles [ti] or abstracts [ab] by adding the corresponding field tags to each keyword “Niemann–Pick type c [tiab] OR Niemann–Pick type c1 [tiab] OR Niemann–Pick type c2 [tiab] OR npc1 [tiab] OR npc2 [tiab]”. Sky blue and green lines indicate original articles and reviews of this subset, respectively.

NPC is a prime example for a disease that lacks a curative therapy despite impressive breakthroughs within the last decades [14,15,16,17] and rapidly growing publication counts (Appendix A, Figure 1). At first sight, the disease seems relatively easy to study and to understand: previous research showed that it is monogenic with autosomal-recessive inheritance and caused by mutations in either of two genes, *NPC1* (OMIM #257220) [18] or *NPC2* (OMIM #607625) [19,20]. The structures of the corresponding proteins [21,22,23,24,25] together with a wealth of cell-based data indicate that this duo collaborates to pilot unesterified cholesterol out of the endosomal–lysosomal system [26,27,28]. If the activity of the membrane-resident NPC1 or its intralumenal partner NPC2 is diminished or absent, unesterified cholesterol accumulates in compartments of the endosome-lysosome [29,30] together with other molecules [31,32]. How can this—at first sight well-defined—cellular problem cause havoc in humans presenting an enormous variability in disease onset, symptoms, and life span? In fact, NPC disease comprises several forms based on the age at which patients present neurological symptoms [14,20,33,34,35,36,37,38]. Rare peri- and neonatal cases present hepatosplenomegaly, jaundice, and fetal hydrops with rapid death often due to hepatic and respiratory failure [39,40,41,42,43,44]. Most patients show infantile forms presenting hypotonia and delayed motor development (early: <2 years) as well as clumsiness, speech delay, and cataplexy (late: 2–6 years) reaching life spans of several years [20,44,45]. The second largest group of patients shows the juvenile form (6–15 years) presenting cognitive impairment, ataxia, and dystonia [20,38,40,41,44]. The adolescent/adult form (>15 years) is characterized by cognitive impairment and psychiatric symptoms such as hallucinations and schizophrenia; the number of these patients is probably underestimated [14,20,46,47,48,49,50]. Notably, there is considerable overlap between the groups with respect to symptoms; many patients present common signs such as ataxia, dysphagia, and vertical supranuclear gaze palsy [20,38,41,44]. However, siblings bearing the same mutations can show distinct forms of the disease [20,33].

The diagnosis of NPC disease is complicated by the heterogeneous clinical presentation and therefore depends on laboratory tests. This includes the so-called filipin test, the detection of plasma biomarkers [51,52,53,54,55], and genetic analyses [56,57,58]. For decades, the filipin test represented the sine qua non to diagnose NPC. It requires primary cultures of fibroblasts from patient-derived skin biopsies followed by the staining of chemically fixed cells with filipin. This bacteria-derived, fluorescent complex of molecules binds unesterified cholesterol, thus allowing the visualization of its intracellular distribution [59]. Therapeutic options are limited to symptomatic treatment [14]. The only disease-modifying drug approved for NPC in many countries, except for the USA, is Miglustat/Zavesca (*N*-butyldeoxynojirimycin), which decelerates disease progression in some patients [38,60,61]. The drug also serves as FDA-approved substrate reduction therapy for Gaucher disease [62,63].

Understanding the somewhat mysterious links between cellular damage and the unpredictable outcome in patients, and the development of diagnostic tests and of efficient therapies require appropriate experimental approaches and models. In the following, we will make some general remarks, we will present currently available models for NPC research, and we will highlight crucial points.

## 2. The Use of Experimental Models in Biomedicine

The use of experimental models in research has a long history. The first “publication” dates to the 17th century, when William Harvey described physiologic experiments with animals such as shrimp, eel, chick, and pigeon to understand blood circulation [64]. For centuries, it was believed that animals are unable to feel pain and that they resemble machines [65]. These views changed during the age of enlightenment: In 1789, the philosopher and jurist Jeremy Bentham was one of the first to raise the issue of animal protection by stating: “The question is not, Can they reason? or Can they talk? but, Can they suffer?” [66]. In 1876, the parliament of the United Kingdom passed the “Cruelty to Animals/Anti-Vivisection Act” that updated previous legislation and imposed rules on experiments with animals. The 20th century saw the establishment of three rules, named replacement, reduction, and refinement (the 3Rs) to match “the intimate relationship between humanity and efficiency in experimentation” [67]. These rules have become a key element of laws regulating the scientific use of animals worldwide [68].

Today, biomedical research on human diseases depends entirely on experimental models that range from single cells to non-human primates. Disease models may emerge from spontaneous changes. A famous example is the nude mouse (*Mus musculus*) introduced by Flanagan [69] and used extensively to create models requiring immunodeficiency, for example for patient-derived xenografts [70]. Models can also be based on healthy animals, in which a disease-like state is induced experimentally. Examples include pharmacologically-induced diabetes in rodents and rabbits [71,72], Parkinson-like symptoms in non-human primates [73] and autism-like behavior in rats (*Rattus norvegicus*) [74]. Other pathologic conditions such as stroke and retinal ischemia can be provoked by an artificial interruption of blood supply [75] and increase of intraocular pressure [76,77], respectively. Loss of bone mass mimicking osteoporosis occurs after tail immobilization in rats [78]. Meanwhile, most experimental disease models are generated by powerful genetic tools. Not surprisingly, oncology was the first area profiting from genetically modified mice with transgenic expression of oncogenes [79]. Mice are not the only species used to mimic human pathologies. The nematode *Caenorhabditis elegans* has been genetically modified to generate models of Parkinson’s [80], Alzheimer’s disease [81], PolyQ disease [82], and lysosomal disorders [83]. The fruit fly *Drosophila melanogaster* serves as disease model for different organs including the brain [84,85,86], kidney [87], and pancreas [88].

The usefulness of a model depends on the specific question. Ideally, the model accurately recapitulates key aspects of the disease of interest, for example pathologic changes in cells or symptoms of patients. Furthermore, it should allow extrapolating results to the target organism. Interestingly, history teaches that extrapolability does not necessarily scale with evolutionary kinship: closer may not be better. For example, thalidomide and aspirin are well tolerated by mammalian species but not by pregnant women [89], and chimpanzee have proven inappropriate for studies on AIDS [90]. Similarly, body size and metabolic rates do not always scale with disease processes. For example, some drugs can be effective at different dosages in different animal models [91,92] and humans [93]. In addition, some animals simply do not show specific symptoms: rats cannot really cough [94], rabbits (*Oryctolagus cuniculus*) and rats do not show some symptoms of cystic fibrosis [95], and no animal except for non-human primates displays endometriosis symptoms [96]. On the other hand, exotic species can serve as important models for human diseases. Examples are the armadillo *Dasypus novemcinctus* for research on leprosy [97], the turtle *Trachemys scripta* to study brain hypoxia and anoxia [98], and the pet *Chinchilla lanigera* to investigate hearing loss [99]. Diurnal rodents represent unique models of cone-related retinal diseases [100].

Disease models based on cultured cells have seen a remarkable renaissance due to the possibility of generating specific human cells from patient-derived induced pluripotent stem cells [101]. A recent article exemplifies this new approach going from in vitro data to retrospective analysis of clinical data exposing a possible treatment [102].

## 3. Experimental Models for Niemann–Pick Type C1 and C2 Disease

**Figure 2:**
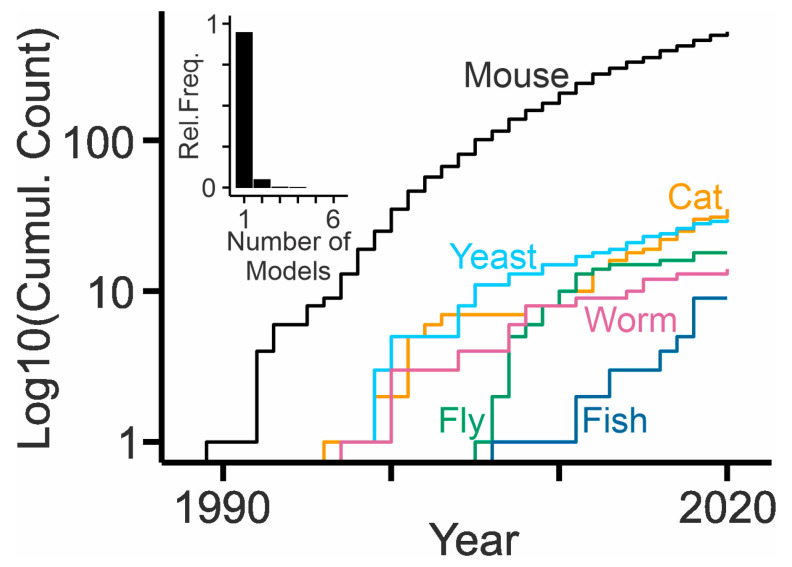
Use of experimental models in NPC research. Cumulative counts (log10 values) of publications obtained by respective Boolean queries in PubMed [e.g., for mouse: (Niemann–Pick type c [tiab] OR Niemann–Pick type c1 [tiab] OR Niemann–Pick type c2 [tiab] OR npc1 [tiab] OR npc2 [tiab]) AND (mice [tiab] OR mouse [tiab] OR mus musculus [tiab]) NOT review]. Inset, the histogram shows that most publications relate to one animal model and that only a small fraction of articles contributes to multiple cumulative counts.

Numerous experimental models are available to study NPC disease [103], probably because the disease is monogenic, the transmission is recessive, and orthologues of the causative genes are present in many phyla ranging from plants to mammals [104] (Table 1). The models have driven the enormous progress in the field during the last decades. Most of them concern *NPC1*, which is mutated in 95% of patients. Only a few experimental models are available to study mutant *NPC2*. The presence of multiple isoforms in specific phyla suggests important and so far undiscovered functions of these proteins. Figure 2 indicates the use of the different models based on the number of publications (Appendix A). Clearly, the mouse has become the preferred workhorse in the NPC disease field.

### 3.1. Non-Mammalian Models

The knock-out of *NPC1* orthologues in plants (*At1g42470*, *At4g38350*; *Arabidopsis thaliana*) [105] and yeast (*NCR1*; *Saccharomyces cerevisiae*) [106] have been generated. Because both showed changes in sphingolipid, but not sterol metabolism, and because *NPC2* orthologues were not known [107], it was assumed that the proteins have distinct roles across phyla. However, yeast cells bear a homologue of *NPC2*, which can replace the human version [108]. Moreover, yeast cells have been used to screen for pathways influencing the outcome of *NPC1* deficiency [109,110,111] and to explore the molecular mechanism of sterol transfer based on structural data [25]. The knock-down of *NPC1* and *NPC2* homologues in the sterol auxotroph pathogen *Entamoeba histolytica* revealed their contribution to cholesterol uptake (*Ehnpc1*, *Ehnpc2*) [112]. The genome of *Caenorhabditis elegans* contains two homologues of mammalian *NPC1* (*ncr-1*, *ncr-2*). Elimination of both forms stalls a specific phase of larval development, which is probably due to defects in the intracellular transport of cholesterol and the production of essential steroid hormones [113,114,115] (Table 1). The defects can be rescued by human NPC1L1 and NPC1 proteins [116] and by specific glycosphingolipids and endocannabinoids [117,118].

The elimination of *Npc1a*, one of two *NPC1* homologues from *Drosophila melanogaster*, causes larval lethality (Table 1), which can be rescued by dietary supply of the steroid hormone ecdysone or by local expression of *Npc1a* in the ring gland [119,120]. The elimination of *Npc1b* also causes larval lethality due to defects in sterol absorption in the midgut (Table 1), which cannot be rescued by ecdysone [121]. The fruit fly bears a family of eight genes resembling *NPC2*. The simultaneous elimination of two of these genes, *Npc2a* and *Npc2b*, causes larval lethality and neurodegeneration, which again can be rescued by dietary cholesterol or ecdysone [122,123]. A genetic screen for pathways mediating cholesterol trafficking and steroidogenesis in *Drosophila* revealed that the activation of autophagy can overcome cholesterol accumulation due to NPC1 deficiency [124].

Induced models of NPC were created in the zebrafish *Danio rerio* (Table 1) using anti-oligonucleotide-based knock-down of *npc1* [125,126]. These manipulations interfered with gastrulation and led to the premature death of embryos, which could be rescued by mouse *Npc1* and in part by steroids [125]. Moreover, morpholino-based knock-down mimicked thrombopenia observed in human patients possibly due to defects in myeloid development [126]. CRISPR-CAS-induced null alleles of *npc1* caused premature death with only a few animals surviving into adulthood. Mutant animals showed massive cholesterol accumulation and defects in the liver, cerebellum, and lateral line organ causing disturbed balance and motor control [127,128].

The non-mammalian models clearly matter, as they reveal how functions of NPC-related proteins evolved, they enable screens to identify NPC1- or NPC2-related pathways and processes, and they help to explore new therapeutic approaches. Up to now, their publication counts are lower than those of mammalian models (Figure 2).

### 3.2. Mammalian Models

Of all mammalian species serving biomedical research, only mice and cats (*Felis catus*) are currently used to study NPC (Table 1). No rat or large animal model has been established for this disease. A single case report described NPC-like symptoms in a Boxer dog [149], and a recent study described calfs (*Bos taurus*) with progressive neurologic symptoms due to mutant *NPC1*, suggesting the possibility of a bovine model [144] (Table 1).

The first mouse strains to study NPC carried spontaneous mutations in *Npc1*, namely the insertion of a retroposon (*Nih* allele, further referred to as *Npc1^Nih^*) [18,129] and of a 43 base-pair (*spm* allele, *Npc1^spm^*) [130,132] in BALB/c and C57BLKS/J colonies, respectively each causing a de facto *Npc1* knock-out. These mice present a relatively early onset of the disease, which is characterized by hepatomegaly, weight loss, disturbed motor coordination, tremor, and ataxia. The mice die prematurely between 11 and 13 weeks of age (Table 1). Cells show an accumulation of unesterified cholesterol, gangliosides, and other lipids in different organs and tissues [132,150,151,152,153,154]. A similar phenotype was observed in a genetically modified mouse from the Goldstein/Brown lab. In this line (*Npc1^pf^*), a double mutation (P202A/F203A) abolishes cholesterol binding by NPC1 and invalidates its function, but it leaves its level and localization unaffected [131] (Table 1). The mice discussed so far represent one end of the model spectrum as they lack the NPC1 function completely and irreversibly. The complete absence of NPC1 occurs only in a small fraction of patients [45]. Nevertheless, these models mattered, as they enabled important discoveries including the gene responsible for the disease [18], the progressive neurodegeneration in the cerebellum [155,156,157], and links to autophagy [158,159,160] and Alzheimer’s disease [161]. Moreover, they were used extensively to explore new therapies (Table 2).

More common *NPC1* mutations in humans induce errors in the structure of the protein leading to its degradation but leave its function more or less intact. Mouse models mimicking these changes have appeared on the scene within the last ten years (Table 1). Maue and colleagues described a mouse line with a D1005G variant that was generated by ethyl nitrosourea mutagenesis (*Npc1^nmf164^*) [132]. Praggastis and colleagues presented a knock-in of the human I1061T version of *NPC1 (Npc1^I1061T^*) [133]. This model matters as it represents approximately 20% of all NPC cases [211,212]. The mouse strains bear misfolded NPC1, causing a partial loss of function. The onset of the disease is delayed, its progress is less severe, and the life span is extended to 17 weeks compared to the complete loss-of-function mutants [133] (Table 1). In 2017, two mouse strains bearing specific human mutations were presented together with a thorough characterization of their behavioral phenotypes [134]. The strains carry either an intronic point mutation (c.1554-1009G > A) generating a pseudoexon due to aberrant splicing (*Npc1^Imagine^*) or the c.1920delG mutation, generating a truncated protein (*Npc1^Pioneer^*) (Table 1). Homozygous *Npc1^Imagine^* mice and compound heterozygous animals *Npc1^Imagine/Pioneer^* displayed symptoms similar to those reported in other NPC animal models with an onset of first neurologic symptoms between 7 weeks and an average life span of 9 weeks. Notably, most homozygous *Npc1^Pioneer^* mice died during the embryonic stage; the few surviving mice (1–2%) were predominantly female [134]. The latest entry in the defilé of models bears a mutant *Npc1* allele generated by the CRISPR-Cas technique (*Npc1^em1Pav^*) [135] (Table 1). These mice help address a key question in the field: which factors determine the enormous phenotypic variability observed in patients? Humans with the same mutation can present completely different disease onsets, progress, and life spans [213,214]. In mice, the outcome of a given mutation varies with the genetic background of strains [37,135,215,216,217,218]. Numerous double mutant mice have been created to test whether and how specific candidate genes impact the disease [163,173,177,190,191,219,220,221,222,223,224,225,226,227,228,229,230,231,232,233,234,235]. Sex-dependent differences in behavior [236], life span [37,134], and responses to immune activation [237] and to potential therapies [171,238] were reported in some NPC1 mutant mice, raising the question of whether sex is a modifying factor in NPC disease [37] as in other cholesterol-related pathologies [239,240,241,242] and normal cholesterol homeostasis [243,244].

Several mouse models were established to study the relevance of NPC1 in specific cell types or tissues (Table 1). Using morula aggregation, so-called chimeric mouse lines were generated, in which distinct ratios of cells harbor the wild-type or the mutant allele [158]. Mice for the cell-specific elimination of *Npc1* were based on the Cre/loxP technique (*Npc1^tm1.1Apl^*) [136,245,246] (Table 1). A first study showed that the elimination of *Npc1* from Purkinje cells induces their degeneration but leaves the life span of mice unaffected [136]. A mouse model to study NPC1 deficiency in the liver forgoing neurologic complications was established by intra-peritoneal injections of antisense oligonucleotides in healthy BALB/c mice [137,186]. The over-expression of *Npc1* in specific cell types has been accomplished using classic transgenic mice to target GFAP-expressing cells [138], the inducible TetOn/Off system, which was used to target neurons [139], and the Cre/loxP system allowing the cell-specific reversal of a *Npc1* knock-out [140] (Table 1). These mice enable a cell- or tissue-specific rescue of NPC1 deficiency [218,247,248]. For example, the re-establishment of *Npc1* expression in the liver rescued liver disease, but it did not prevent progressive neurodegeneration and premature death [140]. The use of cell-specific promoters requires a thorough validation of their expression patterns [249,250]. Moreover, the observation that NPC1 deficiency in neurons is sufficient to induce their death [158,245] does not exclude a demise-provoking contribution by non-neuronal cells such as microglia or astrocytes [251,252,253], serving potentially as therapeutic targets.

Compared to *Npc1*, the line-up of mouse models targeting *Npc2* is much smaller. The first mouse line was created by gene targeting, resulting in 4% of normal protein levels. These animals showed a similar phenotype as NPC1-deficient mice and as mice lacking both proteins. The latter finding provided first evidence for the functional cooperation between NPC1 and NPC2 in vivo [145]. Additional lines targeting *Npc2* have been generated using the gene trap approach [146,147] (Table 1). The over-expression of *Npc2* in the liver was accomplished using transgenic mice and specific promoter elements [148]. More mutant alleles of mouse *Npc1* and *Npc2* are listed on the MGI website.

NPC-like symptoms in a domestic cat (*Felis catus*) were first reported by Lowenthal and collaborators [141] (Table 1). A colony was subsequently established, and the cats were further characterized. They develop neurologic symptoms such as ataxia and vestibular defects at juvenile age similar to humans, and they show neuroaxonal dystrophy [141,142,254,255,256,257,258]. In 2003, the genetic defect was uncovered: a single base substitution (2864G-C) in *NPC1* causes an amino acid change (C955S) [143]. Two case reports described cats with distinct mutant alleles of *NPC1* [259] and *NPC2* [260], indicating that more feline NPC models could be established.

### 3.3. In Vitro Models

**Figure 3:**
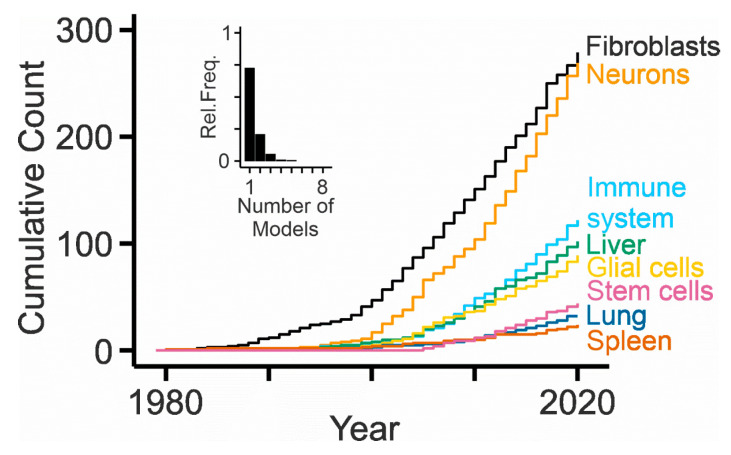
NPC research on specific types of cells. Cumulative counts of publications obtained by respective Boolean queries in PubMed [e.g., for fibroblasts: (Niemann–Pick type c[tiab] OR Niemann–Pick type c1[tiab] OR Niemann–Pick type c2[tiab] OR npc1[tiab] OR npc2[tiab]) AND (fibroblast[tiab] OR fibroblasts[tiab]) NOT review]. Inset, the histogram shows that most publications relate to one cell model and that only a small fraction of articles contributes to multiple cumulative counts.

Cultured cells are instrumental to uncover basic protein functions and molecular disease mechanisms and to test potential therapeutic approaches at the cellular level [12]. The use of cell cultures to study NPC disease dates back to the 1960s, when the Fredrickson group prepared primary fibroblasts from skin and bone marrow of patients with different forms of Niemann–Pick disease, including type C [261]. This pioneering publication initiated a decades-long series of studies based on patient-derived fibroblasts (Figure 3), enabling ground-breaking discoveries. Examples are the defect in cholesterol esterification and the accumulation of unesterified cholesterol [262,263,264], the functional validation of *NPC1*-encoding cDNA [265] and of secreted NPC2 [19], and the degradation of the misfolded p.I1061T NPC1 variant [266].

An alternative method to induce the cellular hallmark of NPC, an accumulation of unesterified cholesterol, relies on hydrophobic amines such as U18666A [267,268,269,270]. Originally, this molecule was developed as an inhibitor of cholesterol synthesis [271], and it was later shown to inhibit NPC1 activity directly [272].

#### 3.3.1. Cell-Lines

The first cell lines to study NPC disease were established from patient-derived blood lymphocytes, which were immortalized through transformation by the Epstein–Barr virus [273]. A similar approach was used to immortalize lymphoid cells from NPC2 patients [153]. A fibroblast cell line based on the *Npc1^spm^* mouse was generated using a spontaneous immortalization (3T3) protocol [274,275]. Immortalized mouse embryonic fibroblasts from NPC1-deficient mice were transduced with different constructs to monitor autophagy [276]. A mouse embryonic fibroblast cell line from NPC2-deficient mice expressing a NPC2–crmCherry fusion protein was established to track the intracellular distribution of the protein [277]. A line of NPC2-deficient patient human fibroblasts showed a down-regulation of NPC1 upon infection with HIV [278]. Several models were derived from Chinese hamster ovary (CHO) cells, the workhorse of cell biology: NPC1-deficient CHO cells were generated using chemical or gene trap mutagenesis and assays to detect cholesterol transport-deficiency [279,280,281]. Other CHO lines stably over-express NPC1 [282,283], myc-tagged NPC2 [284], as well as NPC1-EGFP or -RFP fusion proteins [285,286,287], allowing for example to track the movement of NPC1-containing organelles [285]. CRISPR-Cas technology [288] or transfection with short interfering RNA constructs were used to generate NPC1- and NPC2-deficient HeLa [289,290,291,292] and Hek-293T cells [293]. The knock-down of *NPC1* in a neuroblastoma cell line (SH-SY5Y) was achieved by stable transfection with short hairpin RNA [294]. Immortalized human hepatocytes and hepatic stellate cells with stable knock-down of *NPC1* or *NPC2* were obtained by transduction with lentivirus and short hairpin RNAs [295,296]. The artificial expression of *NPC1* in *Escherichia coli* has been used to study its transport function [297]. In the context of Alzheimer disease research, *NPC1* was stably down-regulated in a neuron-like Neuro-2a line that over-expresses a specific form of the amyloid precursor protein [298]. Schwann cell lines were derived using dorsal root ganglia and peripheral nerves of the *Npc1^spm^* mouse [299]. Knock-down in an oligodendroglial cell line was accomplished using short interfering RNA [200]. The first NPC model based on a haploid human cell line has been introduced recently [300].

Cell line-based models matter to uncover basic molecular functions of NPC1 [21] or NPC2 [301] and NPC1-dependent signaling pathways [293], to perform comparative studies at the cellular level [302], and to identify disease-relevant genes [289]. Cell lines helped to identify NPC1 as a receptor mediating Ebola virus infection [303,304] and to investigate its involvement in hepatitis C virus replication [305]. However, they cannot inform about cell-type specific dependency on NPC1 and consequences of its dysfunction. Moreover, it is not clear whether NPC1- or NPC2-related cellular processes observed in cell lines occur also in specialized cells in vivo. Another caveat derives from the fact that cell lines are per definitionem mitotic, whereas most differentiated cells in the body are post-mitotic. Cell division may modify how NPC1- or NPC2-deficiency affects cells.

#### 3.3.2. Primary Cultures of Brain Cells

An alternative to cell lines are primary cultures, where cells are isolated from the organism and used after different periods of culture without immortalization. Cultured cells retain their in vivo properties to degrees that depend on the cell type and the culture conditions, namely the artificial exposure to chemically undefined serum [306,307,308,309,310].

**Figure 4:**
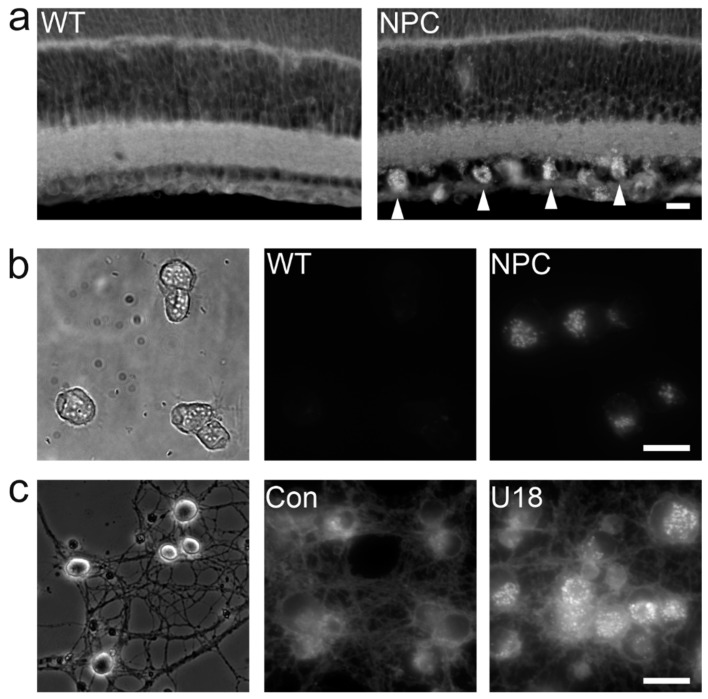
Models to study the impact of NPC1 deficiency on selected neurons in the retina. (**a**) Fluorescence micrographs of retinal neurons from one-week-old wild-type (WT) and NPC1-deficient (NPC) mice in vivo. NPC1 deficiency causes an intracellular accumulation of unesterified cholesterol in neurons of the ganglion cell layer in vivo (arrowheads). (**b**) Phase-contrast (left) and fluorescence micrographs (middle, right) of retinal neurons acutely isolated from one-week-old wild-type (WT: left, middle) and NPC1-deficient mice (NPC). In this ex vivo model, NPC1-deficient neurons maintain the increased levels of cholesterol as shown by filipin staining. (**c**) Phase-contrast (left) and fluorescence micrographs (middle, right) of neurons purified from the retina of one-week-old rats, cultured for 48 h and stained with filipin. Treatment with the NPC1-inhibiting drug U18666A induced an accumulation of unesterified cholesterol. Scale bars: 20 µm. In (**a**–**c**), the distribution of unesterified cholesterol was shown by the staining of chemically fixed material with filipin (**a**,**b**): Barthélémy, Pfrieger, unpublished; (**c**): modified from [317].

Most NPC patients suffer from debilitating neurologic symptoms and therefore, it appears imperative to study the impact of dysfunctional NPC1 or NPC2 on cells in the brain (Figure 3). The first studies using primary cultures of central nervous systen (CNS) cells investigated the expression and distribution of NPC1 in cerebellar neurons and glial cells [311] and reported defects in cholesterol metabolism and neurotrophin signaling in striatal neurons [312]. Thereafter, sympathetic [313], cortical [314], hippocampal [315,316] and retinal neurons [317] (Figure 4) as well as purified cerebellar Purkinje cells [318] have been studied in vitro. These models matter, as they revealed neuron-specific defects caused by NPC1 deficiency such as impaired synaptic function [316,318,319], depletion of cholesterol from axons, and an accumulation of cholesterol independently from lipoprotein uptake [313,317]. They also helped to identify lamellar inclusions as the site of cholesterol accumulation [317]. Cultured astrocytes [320], oligodendrocytes [321,322,323], and microglial cells [324,325] have rarely been studied, despite the potential glial involvement in neurodegeneration [251], evidence for myelination defects [246], and signs of neuroinflammation in NPC disease [326]. Organotypic cultures represent a more integrated preparation to study neurons, but they have been used only sporadically in this field [325,327].

#### 3.3.3. Primary Cultures of Other Cells

Predominant in vitro models of NPC research are the above-mentioned patient-derived skin fibroblasts, which are mitotic primary cells, but not cell lines unless they have been immortalized. Only very few differentiated cell types are studied in the field (Figure 3). Liver and spleen are affected in many NPC patients, but few reports used primary hepatocytes [199,328,329] and hepatic stellate (Ito) cells [330] from NPC1-deficient mice, splenocytes from NPC2-deficient mice [146], and NPC1-deficient splenic B cells [331]. Acutely isolated Kupffer cells were examined in chimeric mice following bone marrow transplantation [332]. With respect to lung defects, one report studied primary type 2 pneumocytes treated with U18666A [333]. With respect to immune cells, studies used NPC1-deficient macrophages [328,332,334], invariant Natural Killer T cells and human B cell lines [335], lymphoblasts [275], monocyte-derived dendritic cells [336], and T cells [146,337,338]. To date, no studies on cultured leukocytes or granulocytes have been published. Among other cells, the effects of NPC2 knock-down on adipocyte differentiation and function were studied using primary cultures [339], and spermatozoa from NPC2-deficient mice were isolated and analyzed [340].

#### 3.3.4. Stem Cell-Derived Models

The differentiation of specific cell types from embryonic or induced stem cells has become popular, because this technology allows studying cells from patients and producing them in large quantities. Consequently, the number of publications related to these models in the NPC field is increasing (Figure 3). A first report showed the impaired self-renewal and differentiation of neural stem cells from embryonic brains of NPC1-deficient mice [341]. Ordonez and colleagues created a short hairpin RNA-based knock-down of *NPC1* in human embryonic stem cells and differentiated these cells to neurons [342]. These neurons recapitulated the pathologic hallmark of NPC, the accumulation of unesterified cholesterol, and showed impaired mitochondrial function and defective autophagy. Multipotent adult stem cells were isolated from skin biopsies of NPC patients and control subjects and differentiated to neurons showing an accumulation of cholesterol [343]. These cells were selected by specific culture conditions. An alternative and meanwhile standard approach is the reprogramming of cells from adult tissues to create induced pluripotent stem cells and their subsequent differentiation to specialized, often postmitotic cells. Several studies used this approach to generate neurons from NPC patients and healthy donors [344,345,346,347,348,349]. Maetzel and colleagues also generated stem cell-derived hepatic cells and isogenic control lines to avoid confounding effects by distinct genetic backgrounds of patients and donors [346]. The stem cell-derived models matter: they enable studying the impact of NPC1 or NPC2 deficiency on differentiated human cells, notably neurons, and to explore new therapeutic strategies [347,350,351]. However, the protocols for reprogramming and differentiation need to be standardized to allow for comparison of results.

## 4. Models Mattering for Therapy Development

Experimental models are indispensable for the preclinical exploration of therapeutic approaches. In the NPC field, cell-based screens for targets and drugs used yeast [109], immortalized embryonic fibroblasts [276] or ovarian granulosa cells from mutant mice [352], human stem cell-derived neurons [347,348,350,351,353,354], mutant CHO lines [355], and patient-derived fibroblasts [356,357]. Numerous therapeutic approaches were tested in vivo using NPC mice and cats. Table 2 lists studies where the impact of treatments on disease progression was assessed with proper controls.

Few studies have delivered an approved drug or treatments reaching clinical development. The disease-modifying *N*-butyldeoxynojirimycin inhibits glucosylceramide synthase [358]. Curiously, a first in vitro study on CHO cells showed that the compound does not revert cholesterol accumulation in NPC1-deficient cells [359]. This was also observed in stem cell-derived neurons in vitro [347], arguing against a therapeutic effect. However, in vivo studies showed that the drug slows down neurologic disease progression and prolongs the life span of NPC1-deficient BALB/c mice and *NPC1* mutant cats [184,360], providing preclinical evidence for its therapeutic use (Table 2).

A potential treatment is based on 2-hydroxypropyl-beta-cyclodextrin (CD) that chelates cholesterol and other components [361] (Table 2). Curiously, the exploration of this compound started with in vivo experiments—again with discouraging results. A first study using intra-peritoneal or intra-thecal injection in NPC1-deficient mice failed to show a positive effect [362]. However, subsequent reports revealed that CD prolongs the life span, slows down neurologic disease progression, and halts the degeneration of Purkinje cells in the mouse and cat model [177,178,179,180,181,363,364]. Intra-thecal injections were required, as CD cannot pass the blood–brain barrier [365]. NPC1-deficient mice were also used to study ototoxicity of CD [366,367] and its effects on microglial cells [368] and the liver [369]. Effects of CD on NPC1-deficient cells were explored in lateral line neuromast cells in vivo [128], siRNA-treated HeLa cells [370], liver-derived cell lines [371], cultured fibroblasts [372,373,374], and primary [317,375,376] or stem cell-derived neurons [342,353,377]. First clinical data showed that CD decelerates disease progression in patients [378,379].

Histone deacetylases (HDACs) emerged as a possible therapeutic target for NPC from a genetic screen in yeast [109] and from in vitro studies of NPC1-deficient neuronal stem cells [380], patient- and mutant mouse-derived fibroblasts [133,357,374,381,382,383,384], cell lines [384,385], and U18666A-treated hippocampal neurons [386]. A first in vivo study using *Npc1* mutant mice claimed that repeated intra-peritoneal injections of vorinostat, an HDAC inhibitor, together with polyetheylene–glycol and CD slow down neurologic disease progression, but some controls were missing [387]. A subsequent report on mice attributed the effects on neurologic symptoms to CD [363]. Repeated intra-peritoneal injections of vorinostat in NPC1 mutant mice improved liver function but did not slow down weight loss or increase life span [194] probably because the drug cannot enter the central nervous system [363]. A comparison of drug effects using different mouse models revealed that drug effects on liver function were not mediated by proteostatic effects on NPC1 [194] (Table 2).

Evidence from *Npc1* mutant mice that heat-shock proteins protect Purkinje cells from degeneration suggested these components as new drug targets in NPC [157,227]. The idea was supported by in vitro studies on patient-derived fibroblasts [193,227,388] and U18666A-treated neurons [227] and in vivo studies exploring the over-expression or knock-down of heat shock protein beta-1 in NPC1-deficient mice [227]. A corresponding disease-modifying therapy may be based on arimoclomol, a small molecule enhancer of heat shock proteins, whose effects were explored in patient-derived fibroblasts and NPC1-deficient mice [193] (Table 2).

Within the last years, NPC1-deficient mice also helped to explore gene therapy for NPC (Table 2). First support for this approach came from two observations. The over-expression of NPC1 in brain cells was achieved following the intra-cerebral injection of an adenoviral construct in vivo [389]. The cell-specific over-expression of NPC1 in transgenic mice rescued pathologic changes due to NPC1 deficiency [139,390]. Within the last few years, a series of studies showed that the progress of neurologic disease in NPC1-deficient mice is slowed down by intra-cardiac [195], intra-cisternal [196], and intra-cerebroventricular [197] injection of vectors based on adeno-associated virus 9 (AAV9). Similar improvements were found in mice lacking NPC2 following intra-cisternal injections of AAVrh.10 carrying NPC2 [198].

## 5. Conclusions and Outlook

The diversity and validity of experimental models and their pertinence to topics of interest are key to advance biomedical research. Over the last decades, the NPC field has developed a gang of models that matter as they revealed the origin of the disease, provided important insight in disease mechanisms, and helped to explore new diagnostic and therapeutic approaches. Moreover, these models are used extensively outside the NPC field to understand fundamental aspects of cholesterol homeostasis [391] in different organs, notably the brain [392], and mechanisms of other cholesterol-related diseases [393,394,395].

The publication record indicates a clear preference for NPC1, mice, and fibroblasts as gene, animal, and cell of choice, respectively. A few points should be considered with respect to future developments and advances. The focus on NPC1 is understandable given that most patients bear mutations in this gene. However, new models targeting NPC2 are of high interest, as they can help for example to discern NPC1- and NPC2-dependent genetic, epigenetic, and sex-dependent disease modifiers. The identification of modifiers remains a top priority in the field. The predominance of mouse models in NPC research is readily explained by the increasing ease of genetic manipulations and the relative cost efficacy. However, mice impose several limitations, notably with respect to their small size and their limited behavioral repertoire [396]. Therefore, new models based on larger mammals including rats are highly desirable last but not least to enable the successful translation of therapeutic approaches into the clinic [6]. There is also a clear demand for inducible/reversible pharmacologic models based on highly selective small molecule inhibitors of NPC1 or NPC2. These approaches would allow for before/after studies and thereby help to discern within-subject variability. The surprising discovery that NPC1 serves as receptor for filovirus entry into cells [303,304] will help to develop such inhibitors and new models.

The focus on fibroblasts originates from their availability through skin biopsies, their ease of maintenance, and their long-standing use as a diagnostic tool. However, studies of patient-derived fibroblasts cannot inform about the outcome of NPC1 dysfunction in highly specialized postmitotic cells such as neurons. Therefore, it is imperative to elucidate how specific cells, namely the most vulnerable, react to defects in NPC1 and NPC2. This will require a combination of preparations allowing to study the same type of cells in vivo, ex vivo, and in vitro (Figure 4) as well as new approaches to analyze mRNA, protein, and lipid content of defined cell types replacing transcriptomic, proteomic, and lipidomic studies of entire organs or tissues. As an example, acutely isolated cells combined with single cell transcriptomics [231] represent a first step that needs to be refined and extended with a focus on vulnerable cells in most affected organs, including the brain, liver, and lung. Cells differentiated from induced pluripotent stem cells represent an alternative although with caveats [397]. Whatever the source of cells, advanced culture systems preserving their three-dimensional arrangement should be considered as well [398,399]. The development of therapeutic approaches for neurologic and psychiatric symptoms faces fundamental hurdles with respect to diagnosis and model validity that are not specific to NPC [400,401].

Clearly, the establishment of new models requires substantial investments and bears risks, but ultimately, all that matters are the models: they are indispensable to expose molecular mechanisms underlying the disease and to develop efficient therapies.

## Figures and Tables

**Table 1:** Animal models available to study NPC disease.

*Species, Gene,* Animal model	Symptom Onset	Life Span	Visceral Symptoms	Neurologic Symptoms	Lipid Accumulation in Tissues	References
*Caenorhabditis elegans* *ncr-1* *ncr-1(nr2022)* *ncr-2* *ncr-2(nr2023)*	ND	Dauer formation	ND	Modified trafficking or release of synaptic vesicles	Nerve ring, spermatheca and oocytes: DHE accumulation	[113,114,115]
*Drosophila melanogaster* *Npc1a* *Npc1a^57A^*	ND	Larval lethality	More efficient sterol absorption than wild-type animals	ND	Malpighian tubules and midgut: Sterol accumulation. Brain and retina: Chol aggregates	[119,120]
*Drosophila melanogaster* *Npc1b* Npc1b^R9-28^	ND	Larval lethality	Defects in sterol absorption; similar to NPC1L1	ND	No Chol accumulation	[121]
*Drosophila melanogaster* *Npc2a* Npc2a^376^ *Npc2b* Npc2b^19^	ND	Larval lethality		Apoptotic cell death in the nervous system	No sterol distribution abnormality	[122,123]
*Danio rerio* *npc1* *npc1^ihb334^* *npc1^ihb335^* *npc1^hg37^* *npc1^y535^*	Larval stage	99% animals die within the first MPF; 1% die before 8 months of age	Hepatomegaly, splenomegaly	Disturbed balance and motor control, loss of Purkinje cells	Liver: accumulation of Chol, CER, DG, LPA, PA, PC, PE, PS, TG, SL	[125,126,127,128]
*Mus musculus* *Npc1* BALB/cNctr-*Npc1^m1n^*/J *Npc1^nih^*	6 wks (N)	9–11 wks	Hepatomegaly, splenomegaly, decreased weight gain, increased lung mass	Disturbed motor coordination, tremor, ataxia, loss of Purkinje cells	Spleen, liver, lung, lymph nodes, thymus, bone marrow, brain: accumulation of FA, CER, Chol, SL	[18,129]
*Mus musculus* C57BLKS/J-*Npc1^spm^*/J *Npc1^spm^*	4 wks (V)	11–15 wks	Hepatomegaly, splenomegaly, decreased weight gain	Disturbed motor coordination, tremor, ataxia, loss of Purkinje cells	Liver: accumulation of FA, CER, Chol, SL. Brain: Chol accumulation	[130]
*Mus musculus* *Npc1^tm1Mbjg^* *Npc1^pf^*	7 wks (N)	12 wks	Hepatomegaly, splenomegaly, decreased weight gain	Tremor, ataxia, loss of Purkinje cells	Brain, kidney, liver, lung, and spleen: Chol accumulation. Brain and liver: GM accumulation.	[131]
*Mus musculus* *Npc1^nmf164^*/J *Npc1^nmf164^*	4 wks (N)	16 wks	Hepatomegaly, splenomegaly, decreased weight gain, foamy pulmonary macrophages	Loss of Purkinje cells, abnormal acoustic startle response, decreased strength and motor capabilities	Brain: Chol and GM accumulation. Liver: accumulation of CER, Chol, SL, GM.	[132]
*Mus musculus* *Npc1^tm(I1061T)Dso^* *Npc1^I1061T^*	8 wks (N)	17–18 wks	ND Decreased weight gain	Decreased motor coordination, tremor, loss of Purkinje cells	Liver and brain: Chol accumulation	[133]
*Mus musculus* *Npc1^tm1Tacf^*/J *Npc1^Imagine/Imagine^*	7 wks (N)	9 wks	ND	Decreased motor coordination, tremor, ataxia, age-dependent hyperactivity, reduced anxiety, cortico-hippocampal defects, higher pain threshold	ND	[134]
*Mus musculus* *Npc1^tm2Tacf^*/J *Npc1^Pioneer^/^Pioneer^*	ND	Only 2% live births	ND	ND	ND	[134]
*Mus musculus* *Npc1^Imagine^/^Pioneer^*	7 wks (N)	9 wks	ND	Decreased motor coordination, tremor, ataxia, age-dependent hyperactivity, reduced anxiety, higher pain threshold	Liver: Chol and CER accumulation Brain: Chol, CER, GM accumulation	[134]
*Mus musculus* *Npc1^em1Pav^*	4 wks (V,N)	10–12 wks, strain-dependent	ND	Loss of Purkinje cells, decreased motor coordination	Liver, brain, spleen: GM accumulation	[135]
*Mus musculus* *Npc1^tm1.1Apl^* Cell-specific knock-out based on Cre/loxP			Depends on target cells/tissues			[136]
*Mus musculus* *Npc1*(ASO) Knock-down of NPC1 in liver and lung by antisense oligonucleotides (ASOs)			Hepatomegaly; foamy, vacuolated macrophages and increased apoptosis/proliferation in liver	No neurologic symptoms	Liver: Chol accumulation	[137]
*Mus musculus* Tg(Gfap-Npc1) Rescue of Npc1 expression in Gfap-expressing cells	Delayed onset with respect to NPC1^-/-^ (N)	24 wks	Weight gain with respect to NPC1^-/-^	Reduced numbers of axonal spheroids and reactive astrocytes, restoration of myelin, loss of Purkinje cells, decreased neurodegeneration with respect to NPC1^-/-^	Reduced Chol accumulation in some brain areas with respect to NPC1^-/-^	[138]
*Mus musculus* Tg(tetO-Npc1/YFP)1Mps Cell-specific over-expression based on Tet-On/Off			Depends on target cells/tissues			[139]
*Mus musculus* *Npc1^fl/fl^* Cell-specific reversal of Npc1 knock-out based on Cre/loxP			Depends on target cells/tissue			[140]
*Felis catus* *NPC1*	6 wks (N)	20 wks	Hepatomegaly, spleen and lung with multifocal histiocytosis	Tremor, ataxia, loss of Purkinje cells, astroglyosis, myelin abnormalities in peripheral nervous system	Pyramidal neurons: GM2 accumulation	[141,142,143]
*Bos taurus* *NPC1*	3 months (N)	before 8 months (N = 1)	Marked hypertrophy of Purkinje cells in heart, foamy macrophages in lymph nodes	Limb weakness, dysmetria, incoordination, a wide based stance, walking sideways or falling over and recumbency, vacuolation of Purkinje cells, astrocytosis, microgliosis	Fibroblasts: Chol, GM, SL accumulation	[144]
*Mus musculus* *Npc2^tm1Plob^*	4 wks (V)	18 wks	Decreased weight gain	Tremor, motor defects, ataxia, loss of Purkinje cells	Liver: Chol accumulation, neocortex, dentate gyrus, hippocampus, and cerebellum: Chol accumulation	[145]
*Mus musculus* *Npc2^Gt(LST105)Byg^*	8 wks (N)	ND	Decreased weight gain	Tremor, ataxia, loss of Purkinje cell, astrocytosis	Liver, spleen, kidney, lung: Chol accumulation.	[146,147]
*Mus musculus* Tg(Apoe-Npc2) Over-expression of NPC2 in liver	ND	ND				[148]

Abbreviations: not determined (ND); weeks (wks); neurological symptoms (N); visceral symptoms (V); cholesterol (Chol); ceramide (CER); dehydroergosterol (DHE); diacylglycerol (DG); gangliosides (GM); lysophosphatidic acid (LPA); months post fertilization (MPF); phosphatidic acid (PA); phosphatidyl–choline (PC); phosphatidyl–ethanolamine (PE); phosphatidyl–serine (PS); sphingolipids (SL); triglycerides (TG)

**Table 2:** Summary of therapeutic approaches for NPC explored with animal models.

Treatment	Model	Effect	Reference
Cholesterol lowering drugs	*Npc1^nih^*	No	[162]
Apoptosis, inhibition	*Npc1^nih^*	No	[163]
Mitogen-activated protein kinase, inhibition	*Npc1^nih^*	No	[164]
Dietary restriction	Cat	No	[165]
Implantation of neural stem cells	*Npc1^nih^*	No	[166]
Transplantation of mesenchymal stem cell	*Npc1^nih^*	Small	[167,168,169]
Vitamin C	*Npc1^nih^*	No	[170]
Vitamin E	*Npc1^nih^*	Yes	[171,172]
Liver X receptor, activation	*Npc1^nih^*	Yes	[173]
Pregnane X receptor, activation	*Npc1^nih^*	Yes	[174]
Estradiol	*Npc1^nih^*	Small	[175]
C-Abl inhibition (Imatinib)	*Npc1^nih^*	Yes	[176]
2-hydroxypropyl-beta-cyclodextrin	*Npc1^nih^*, cat	Yes	[177,178,179,180,181]
Cyclin-dependent kinase-5, inhibition	*Npc1^nih^*	Small	[182]
Non-steroidal anti-inflammatory drugs	*Npc1^nih^*	Yes	[170]
Protein replacement, NPC2	129P2/OlaHsd-*Npc2*^Gt(LST105)BygNya^	Small	[147]
Curcumin	*Npc1^nih^*	No	[183]
Glucosylceramide synthase, inhibition	*Npc1^nih^*, cat	Yes	[184,185]
N-acetylcysteine	*Npc1^nih^*, *Npc1*(ASO)	Small	[186]
Copper chelation	*Npc1^nih^*	Yes, not CNS	[187]
Acetylcholinesterase, inhibition	*Npc1^nih^*	Small	[188]
Combination miglustat, curcumin, ibuprofen	*Npc1^nih^*	Yes	[189]
Glucocerebrosidase, inhibition	*Npc1^nih^*	Yes	[190]
Necroptosis, inhibition	*Npc1^nih^*	Yes	[191,192]
Heat shock protein, activation (Arimoclomol)	*Npc1^nih^*	Yes	[193]
Histone deacetylases, inhibition (Vorinostat)	*Npc1*^nmf164^, *Npc1^nih^*	Yes, not CNS	[194]
Gene therapy, AAV9-NPC1	*Npc1^nih^*	Yes	[195,196,197]
Gene therapy, AAV rh.10-NPC2	*Npc2^tm1Plob^*	Yes	[198]
Glutathion	*Npc1^nih^*	Yes	[199]
Adenosine A2A receptor, activation	*Npc1^nih^*	Yes	[200]
Polymeric beta-cyclodextrin	*Npc1* ^nmf164^	Small	[201]
Pneumococcal immunization	*Npc1^nih^*	Yes	[202]
Histamine H3 receptor, activation	*Npc1^nih^*	No	[203]
6-O-alpha-maltosyl-beta-cyclodextrin	*Npc1^nih^*	Yes	[204]
Implantation of VEGF-overexpressing neural stem cells	*Npc1^nih^*	Yes	[205]
CYP46A1, activation	*Npc1* ^nmf164^	Yes	[206]
High-density lipoprotein nanoparticles	*Npc1* ^I1061T^	Small	[207]
Gene therapy, AAV-mediated base editing	*Npc1* ^I1061T^	small	[208]
Iron chelation	*Npc1^nih^*	No	[209]
Gene therapy, Trojan horse liposomes	*Npc1^nih^*	No	[210]

## Appendix A

To obtain quantitative data on the publication output in the field, Boolean queries were performed in PubMed and records were downloaded in csv format. Data analysis and visualization were accomplished using the open source software R [402] and selected R packages (data.table [403], ggplot2 [404], readr [405].
